# Rubella Vaccine Uptake among Women of Childbearing Age in Healthcare Settings

**DOI:** 10.3390/healthcare11222992

**Published:** 2023-11-19

**Authors:** Cristiana Ferrari, Giuseppina Somma, Sandro Gentili, Gianmarco Manili, Gaetano Mauro, Michele Treglia, Marco Trabucco Aurilio, Andrea Magrini, Luca Coppeta

**Affiliations:** 1 Department of Biomedicine and Prevention, University of Rome Tor Vergata, 00133 Rome, Italy; 2 Office of Medical Forensic Coordination, Italian National Social Security Institute (INPS), 00144 Rome, Italy

**Keywords:** rubella vaccine, rubella, vaccination strategy, immunity for rubella, hesitancy, vaccine acceptance

## Abstract

Background: Rubella is a contagious viral infection that occurs most often in children and young adults. Rubella is the leading vaccine-preventable cause of birth defects. Rubella infection in pregnant women may cause fetal death or congenital defects known as congenital rubella syndrome. There is no specific treatment for rubella, but the disease is preventable by vaccination with an efficacy of over 95%. Vaccination coverage is still below the recommended levels and many cases have occurred worldwide. The COVID-19 pandemic has had a negative impact on the immunization programs and the quality of disease surveillance worldwide. Operators of the healthcare setting are at increased risk of infection due to their work duties and should receive preventive vaccination or serologic protection to work in a healthcare setting. Aims: To evaluate the serological evidence of rubella IgG antibodies in female healthcare operators of childbearing age, to assess the risk of a breakthrough infection and the need for an additional dose of vaccine. Methods: We collected age and antibody titers from 449 young female operators aged <50 years who underwent the periodic surveillance at the Occupational Medicine Unit of the Policlinico Tor Vergata, Rome, from January to July 2022. Subjects were considered immune if the anti-rubella IgG titer was >11.00 IU/mL. Results: The rate of serologically unprotected subjects was 9.13% (41/449). The mean age of protected subjects was 26.93 years, while the mean age of unprotected subjects was 28.24 years. Age did not correlate with mean titer on statistical analysis (*p* = 0.10). The acceptance rate among unprotected operators was 31.7%. A positive attitude towards vaccination was found in 11/28 (39.3%) of the unvaccinated subjects, while a negative tendency was found in 2/28 (7.1%) of these subjects; most of the unvaccinated operators 15/28 (53.6%) prefer to postpone the administration of the vaccine. When compared with a similar population from the pre-pandemic period, the actual proportion of immune female subjects was not significantly different from that found in 2019 (90.87% vs. 90.3%). Conclusions: Protection against rubella was suboptimal among female healthcare workers of childbearing age. Acceptance of the rubella vaccine among these operators was low. Most of those who were hesitant intended to postpone the vaccination, while a minority had negative attitudes toward vaccination. A policy of mandatory vaccination policy should overcome the reluctance of operators.

## 1. Introduction

Rubella is an acute, contagious infection caused by an RNA virus of the genus Rubivirus of the family Togaviridae, which is spread by airborne droplets when infected persons sneeze or cough. Although it is usually a mild illness in adults, with symptoms including rash, low-grade fever, nausea, and mild conjunctivitis, rubella infection during pregnancy can be serious, leading to miscarriage, intrauterine death of the fetus, or severe birth defects (cataracts, congenital heart disease, hearing impairment, and developmental delay) (congenital rubella syndrome, CRS) [1,2,3]. The World Health Organization (WHO) has developed a strategic plan for the elimination of measles and rubella, and the prevention of congenital rubella syndrome. The “WHO European Region strategic plan 2005–2010” aims to ensure significant progress in the elimination of rubella transmission in areas where the disease is expected to be eliminated, such as Europe. In April 2012, the Global Strategic Plan for measles and rubella, the “Measles and Rubella Initiative”, was launched for the period 2012–2020. Although rubella can be prevented by vaccination with the live attenuated measles-mumps-rubella (MMR) vaccine, which is >95% effective, 10798 cases were reported worldwide in 2021 and 208 cases were reported in the European Region between May 2022 and May 2023 [4,5]. The MMR vaccine is safe, inexpensive and provides long-lasting protection. However, global vaccination coverage is still below the level recommended by the WHO to ensure elimination of the disease [6]. The CDC (Center for Disease Control and Prevention) reports that about 30% of the world’s children are unprotected against rubella, although the virus has been eliminated in 80 countries, unfortunately not in Italy [7,8,9,10]. The COVID-19 pandemic negatively affected immunization programs and the quality of disease surveillance in many countries around the world. Access to immunization centers and services was reduced due to travel restrictions imposed on the population by total or partial closures to contain the spread of SARS-CoV-2 and to the reduction in the number of MMR vaccine doses administered because of the priority given to the SARS-CoV-2 immunization campaign [11,12]. It is recognized that Health Care Workers (HCWs) are at higher risk of rubella infection than the general population because of the nature of their work [13,14]. Therefore, the Advisory Committee on Immunization Practices (ACIP) has recommended rubella vaccination for these workers [15]. In 2017, the Italian National Plan for Immunization and Prevention established that all HCWs should have presumptive evidence of immunity to rubella (serologic evidence of protective antibodies or documented vaccination with at least one dose of rubella-containing vaccine) [16]. Although two doses of MMR vaccine have been shown to provide long-lasting immunity, the debate regarding the clinical significance of breakthrough infection occurring in individuals with low detectable antibody levels remains controversial [17]. Failure of rubella vaccination is rare and may be due to maternal antibodies interfering with the infant’s immune response, or an incomplete vaccination schedule, or declining antibody levels long after the last dose [18]. Reports of clinical reinfection in vaccinated individuals resulting in fetal damage are rare, although approximately 10% of individuals have no detectable antibody levels 12 to 15 years after receiving the second dose, reflecting the presence of long-lasting cell-mediated immune memory [19]. In women of childbearing potential (the period from puberty to menopause when women can become pregnant and have children), the occurrence of subclinical viremic reinfection, should also be carefully considered because of the potential for trans-placental transmission of the disease to the fetus, resulting in CRS [20]. Based on these considerations, the ACIP recommended an additional dose of MMR vaccine for women of childbearing potential who have received 1 or 2 doses of rubella-containing vaccine but do not have a protective level of rubella IgG antibodies [9]. Suboptimal vaccination rates for vaccine-preventable diseases among healthcare workers have been demonstrated in seroprevalence studies, primarily due to declining antibody titers over time [21,22,23,24,25]. The purpose of the present study was to evaluate serologic protection against rubella in female healthcare operators of childbearing age to assess the risk of a breakthrough infection and the need for an additional dose of vaccine as recommended by the ACIP.

## 2. Materials and Methods

We collected the serologic data from 449 young female operators (nurses, residents, and medical students in training) aged <50 years, from the University Hospital of Rome Tor Vergata, in order to evaluate the level of anti-Rubella antibodies. The physicians were not tested for rubella, because they had already been tested in the past, at the time of their first occupational screening, and, if unprotected, were eventually vaccinated. Data were collected from the records of the subjects who underwent the periodic surveillance visit at the Occupational Medicine Unit during the period January–July 2022. All subjects underwent venipuncture of the cephalic vein, median cubital vein, or basilic vein to collect blood for routine blood testing.

A 10 mL blood sample was collected and sent to the laboratory for the serological evaluation of the anti-rubella IgG in the operator’s serum using a chemiluminescent assay (LIAISON^®^ Rubella IgG, DiaSorin S.p.A., Macquarie Park, Australia), with sensitivity and specificity of 97% (95% CI: 91.7–99.4) and 93% (95% CI: 82.5–97.7), respectively. Age and antibody titer were recorded for each subject. The sample was then stratified into two groups: susceptible and non-susceptible to rubella virus infection. According to ACIP, subjects were considered immune if the anti-rubella IgG titer was >11.00 IU/mL. In women not of childbearing potential, a titer < 11.00 IU/mL was also considered protective if they had received at least one dose of rubella-containing vaccine. Women of childbearing age with a titer < 11 were considered unprotected and therefore susceptible to receiving an additional dose of MMR vaccine. All operators identified as unprotected by the above criteria were offered MMR vaccination.

Acceptance rates and intentions to receive the vaccine were then recorded by telephone interview. The protection rate was compared with the results of a previous survey of a similar population carried out in the pre-pandemic period of COVID-19. The Policlinico Tor Vergata does not have a compulsory vaccination policy for rubella, but operators who refuse vaccination, after having had a dialogue with the occupational physician, who explains the risks and benefits of the MMR vaccine, are simply excluded from direct assistance to rubella patients.

For statistical analysis, quantitative data were presented as mean ± SD (standard deviation), while categorical variables (work task and attitudes toward vaccine acceptance) were presented as number (percentage) of participants. The Chi-square test was used to compare categorical outcomes, and the T-test was used for continuous variables (age and antibody titer). Statistical significance was set at a *p* value threshold of less than 0.05. Analyses were performed using IBM SPSS software (version 23).

## 3. Results

Main population characteristics are listed in Table 1. Clinical records of 449 female operators (nurses, residents, and medical students) were evaluated. The mean age of the study population was 27.05 ± 7.15 years (range 18–50 years). The rate of serologically unprotected subjects (rubella IgG titer < 11.00 IU/mL) was 9.13% (41/449). The mean antibody titer of the entire sample was 51.48 ± 45.60 IU/mL (range 0–401 IU/mL). The mean age of subjects with an antibody titer greater than 11.00 IU/mL was 26.93 years, while the mean age of unprotected subjects (IgG titer < 11.00 IU/mL) was 28.24 years. Statistical analysis with linear regression showed no significant correlation between age and antibody titer (*p* = 0.10). We subsequently identified two age groups, older and younger than 24 years. The positive titer was present in 90.3% of subjects over 24 years and in 89% of subjects under 24 years (*p* = n.s.).

The second phase of the study involved evaluating, from the cohort of 41 women who lacked sufficient antibody protection and were therefore deemed “unprotected”, those who would agree to be vaccinated. MMR vaccination is recommended for all healthcare workers who are serologically susceptible. We also wanted to verify the level of vaccine uptake according to the indications of the Occupational Medicine Unit of the Tor Vergata Policlinico.

Among subjects without adequate antibody coverage, the acceptance rate was 5/12 (41.7%) for nurses, 1/5 (20%) for residents, and 7/24 (29.2%) for students (*p* = 0.53, at Chi-square test). We collected attitudes toward vaccine acceptance among those who refused the booster and classified them into three levels of hesitancy: 11 were for vaccination, two were against vaccination, and 15 decided to postpone vaccination. Of those who favored vaccination, five said they did not know if they were serologically protected against rubella, and only one said he had been advised against vaccination by a physician. Of those who chose not to be vaccinated, one reported that he did not think the vaccine was necessary. Of those who decided to postpone vaccination, one did not respond to a previous vaccination schedule, and two reported having other priorities. Of those who agreed to be vaccinated, four reported that they had already postponed the vaccination, one felt it was very important to be vaccinated, and four did not know that they were not protected against rubella. Comparing the actual rate of immune female subjects (90.87%) with the value of a similar population studied in 2019 (90.30%), we found no significant differences.

The main results are presented in Table 2.

## 4. Discussion

The results of our study confirm the potential risk of rubella infection in young female HCWs due to waning serologic protection in the years following MMR vaccination. In the general population, two doses of MMR vaccine are considered sufficient to provide durable immunity against clinically relevant rubella infections, even in the presence of waning humoral immunity [6]. Therefore, administration of an additional dose is not recommended. Conversely, this recommendation cannot be extended to female healthcare operators because of the low risk of CRS following breakthrough infection during pregnancy [20,26,27]. These workers are considered to be at a higher risk of infection than the general population due to their work activities involving close contact with potentially infected patients. Medical students and trainees may also be at risk [28]. Based on this evidence, ACIP, but not the Italian national recommendations, suggests that additional doses be administered to vaccinated but serologically unprotected female operators [20]. Our findings confirm the need to reevaluate vaccination policies for these operators worldwide. Although reported cases of rubella have declined slightly in Europe in recent decades [29], the presence of asymptomatic infection cannot be excluded because breakthrough rubella is very rarely recognized. Administration of a booster dose may be an effective measure to maintain a protective antibody titer in at-risk operators. In our study, the percentage of serologically protected operators was lower than the expected immunity rate, considering that the vaccination coverage of the Italian population is well above 95%, even more than ten years after two vaccine doses. The decline in humoral immunity, which has been widely reported in recent studies, may explain this lower rate [30].

Although vaccine-induced rubella antibody levels may decline over time, data from rubella and CRS surveillance suggest that immunity does not decline, and susceptibility to rubella does not increase. Approximately 91–100% of individuals who have received two doses of the vaccine have detectable antibodies 12–15 years after the second dose. Previous studies have reported decreasing antibody levels without a loss of immune protection [19,31]. Breakthrough infections in vaccinated individuals are generally mild in nature. However, it is recommended that women of childbearing age be tested for rubella IgG regardless of their vaccination status because it cannot be ruled out that subclinical rubella infection in serologically unprotected women may be associated with maternal-fetal transmission. Therefore, ACIP recommends that these women receive an additional dose of rubella-containing vaccine [20].

Regarding the vaccine acceptance rate, we found a suboptimal vaccine acceptance attitude, especially among medical students and nurses (29.2% and 41.7%, respectively). These findings are consistent with previously reported surveys of influenza and COVID-19 vaccine hesitancy [32,33,34]. The determinants of their hesitancy have not been investigated and therefore remain speculative. We can only make assumptions based on previous studies of COVID-19 vaccination. Differences in vaccine acceptance have been observed between professional groups for different vaccines, with nurses in particular being less likely to accept vaccines than physicians in many of the studies. The predominance of women in the nursing and medical student professions may explain the observed differences. Previous studies have shown that women are less likely to be vaccinated [35,36]. Regarding students, they are a unique subpopulation with different factors and habits, such as a different mindset. Recent experience with COVID-19 has shown acceptance rates similar to those found in our study. Incomplete knowledge of pathogens and immunological mechanisms, especially among younger people, may be a reason for increased hesitancy. Indeed, studies in the literature show that lower levels of education is associated with lower acceptance. They may also be more influenced by misinformation spread by social media and by parental attitudes against vaccines [37,38].

The WHO Strategic Advisory Group of Experts (SAGE) defines vaccine hesitancy as a delay in accepting or refusing vaccination despite the availability of a vaccine [39,40,41] and notes that it is influenced by several factors. The main reasons for HCWs’ vaccine hesitancy are a poor perception of the need for vaccination, with an underestimation of the true severity of the disease (known as complacency); a lack of trust in government, health systems, pharmaceutical companies and the efficacy and safety of vaccines (known as confidence); and difficulties in accessing vaccines (convenience) [42]. The qualitative evaluation conducted in our study adds further knowledge to the general attitude towards the vaccine among these young operators; the majority of HCWs who refused MMR vaccination intended to postpone and/or later accept vaccination, while only two subjects expressed a negative attitude towards vaccination. Understanding the determinants of vaccine acceptance or refusal is crucial for planning specific interventions to increase vaccination coverage among HCWs. Recent experience shows that policies of compulsory vaccination and vaccine accessibility (workplace vaccination) are effective in increasing coverage, while educational policies are ineffective. Stricter vaccination policies or mandatory vaccination should easily result in higher vaccination coverage among these operators. In addition, workplace vaccination has been shown to be cost-effective among these individuals [43,44,45,46,47,48]. We believe that, even in the workplace, hospital policies based on gender differences in susceptibility to nosocomial infections need to be considered by occupational health practitioners [49].

Our study has some potential limitations: first, it did not take into account the differential risk of exposure; second, further research is needed to explore the potential limitation that may arise from to the unknown type of vaccine received in childhood. In addition, some of the operators included in the study did not have a written vaccination schedule, so we could have included among the unprotected subjects those who were not vaccinated in childhood. In particular, vaccination records were not systematically collected because many students were from European and non-European countries, and part of the study population was unable to provide a written vaccination record.

Another limitation of the study is that we did not examine outcomes of previous pregnancies (or pregnancy plans), which may influence immunity to rubella, as women who want to become pregnant are advised to be vaccinated.

The Italian MMR vaccination campaign started in 1972; moreover, the risk in unvaccinated subjects could be even higher, confirming the importance of our results and the need to strengthen the recommendation of mandatory rubella policies.

It would also have been useful to include in the study data from male healthcare workers, particularly those associated with women of childbearing age, although this was outside the scope of the study. This inclusion could have been of great importance, particularly in relation to the potential risks of breakthrough infections in women (both adequately protected and unprotected) working in various other settings if exposed to (high) titers of infection from their respective partners. As a result, vaccination recommendations may benefit from the inclusion of male healthcare workers. This may be of interest for a follow-up study.

## 5. Conclusions

We found a 9.13% rate of serologically unprotected subjects among female HCWs of childbearing age. Vaccine acceptance among these operators was suboptimal. Most of the hesitant operators intended to postpone vaccination, while a minority of them had negative attitudes towards vaccination. Interventions to improve knowledge of vaccine efficacy among HCWs should be promoted to increase vaccine uptake. Vaccination should be promoted with common tools that address common and specific concerns about each vaccine, targeting HCWs, especially nurses and medical students. Serological assessment of antibody responses to vaccines could be useful to confirm the immune response and increase awareness of vaccine efficacy among hesitant operators. Finally, HCW hesitancy should be overcome through mandatory vaccination policies.

## Figures and Tables

**Table 1:** Main characteristics (age, mean antibody titer) of subjects included in the study population.

		N	Mean	%
Population		449		100
Age (mean ± DS)			27.05 ± 7.15	
Mean antibody titer (±DS)			51.48 ± 45.60	
Rubella IgG titer	<11 IU/mL	41		9.13
	>11 IU/mL	408		90.87

**Table 2:** Main results of the study (mean age of the population by antibody titer, vaccine acceptance rate among unprotected subjects, and attitudes toward vaccine acceptance among those who refused the booster).

		N and Rates	%	*p* Value
Mean age according to antibody titer	<11 IU/mL	28.24 years		0.10
	>11 IU/mL	26.93 years		
Acceptance rate among unprotected (IgG < 11 IU/mL)	Nurses	5/12	41.7	0.53
	Residents	1/5	20	
	Students	7/24	29.2	
Attitudes toward vaccine acceptance of those who refused the booster	In favor of vaccination	11/28	39.3	
	Against vaccination	2/28	7.1	
	Decided to postpone vaccination	15/28	53.6	

## Data Availability

The data presented in this study are available on request from the corresponding author.
